# A Study on Regulating the Residual Stress of Electroplated Cu by Manipulating the Nanotwin Directions

**DOI:** 10.3390/mi15111370

**Published:** 2024-11-14

**Authors:** Gangli Yang, Tailong Shi, Liu Chang, Hongjia Zhu, Dongyu Tong, Wending Yang, Zeyuan Li, Liyi Li

**Affiliations:** 1 School of Integrated Circuits, Southeast University, Nanjing 211189, China; 230238438@seu.edu.cn (G.Y.); 101013381@seu.edu.cn (T.S.); 230239483@seu.edu.cn (L.C.); 220231881@seu.edu.cn (H.Z.); 220231883@seu.edu.cn (D.T.); 220236249@seu.edu.cn (W.Y.); 2 School of Power and Mechanical Engineering, Wuhan University, Wuhan 430072, China; zeyuan.li.20@alumni.ucl.ac.uk

**Keywords:** residual stress, nanotwinned Cu, glass substrate, EBSD

## Abstract

Glass substrate, a new type of substrate with excellent mechanical and electrical properties of glass itself, has great potential to become an ideal platform for heterogeneous integration in chiplet systems for high-performance computing applications. The residual stress of the metal layer generated on the glass surface during the electroplating process is one of the major bottlenecks of glass packaging technologies, resulting in glass-metal layer delamination and glass breakage. This paper demonstrated for the first time a method to regulate the residual stress by manipulating the nanotwin directions of the electroplated Cu. The experimental results show that nanotwins with three different directions (non-directional, vertical, and horizontal) can be manipulated by controlling electroplating conditions (concentration of Cl^−^ and gelatin, stirring speed). The orientations of non-directional, vertical, and horizontal nanotwinned Cu are non-oriented, 110 and 111, respectively. After electroplating, the 111-oriented nanotwinned Cu has the smallest residual stress (39.7 MPa). Annealing can significantly reduce the residual stress of nanotwinned Cu, which has been attributed to the decrease in the geometric necessity dislocation density. 110-oriented nanotwinned Cu had drastic recrystallization, while 111-oriented nanotwinned Cu and non-oriented nanotwinned Cu had only slight recrystallization. After annealing, the residual stress of 111-nt-Cu remains the lowest (29.1 MPa).

## 1. Introduction

The development of artificial intelligence (AI) and 5G communication drives the evolution of semiconductor technologies [1,2,3]. Chiplet technology is considered to be one of the feasible ways to transcend Moore’s Law [3,4]. The low-loss tangent, coefficient of thermal expansion (CTE) match with Si, and dimensional stability of glass itself in large panel manufacturing make glass advanced packaging technology a great choice for heterogeneous integration in chiplet systems. However, the surface of the glass is smooth and the adhesion strength between the glass substrate and the metal layer is poor, and the residual stress formed in the Cu electroplating process can cause glass-Cu delamination or glass cracking [5,6]. Moreover, excessive residual stress causes stress concentration in the electroplating and annealing process, resulting in glass substrate breakage [7], which seriously restricts the large-panel mass production manufacturability of glass packaging. Therefore, how to reduce the residual stress of electroplated Cu is one of the key problems in glass packaging technologies [8].

The current studies of the residual stress in electroplated Cu on the glass substrate are mainly based on the finite element simulation. For example, Benali et al. established mathematical models to describe the thermo-mechanical stress of electroplated Cu and verified the models with finite element analysis and simulation [9]. Demir et al. established a finite element model of electroplated Cu at 55 °C to 125 °C to analyze the stress-strain relationship and verified the thermal stress model through thermal cycling reliability testing [10]. These studies could provide theoretical guidance on how to reduce the thermal stress. For example, the thicker the Cu layer, the greater the thermal stress. However, the target thickness of Cu is usually fixed based on the design rules when the test vehicles were initially designed. Thus, methods to regulate the residual stress of Cu without changing the design rules are needed.

In addition to the thermal stress caused by the temperature changes, the residual stress generated during electroplating and annealing also includes the microscopic residual stress caused by changes in the grain characteristics of the electroplated metal [6,8]. For example, Okoro et al.’s research results show that the change in the microstructure of Cu grains during electroplating would produce stress, and the magnitude of stress is related to the grain characteristics [6,7]. At present, the main methods to reduce residual stress are process optimization, surface pretreatment, post-annealing treatment, and so on [11,12,13]. For example, Rodolpho et al. reduced the residual stress of the coating on a low-strength substrate by increasing the thickness of the cold spray coating [11]. Results from Alessandro et al. show that the residual stress of laser-melted AlSi_10_Mg can be significantly reduced using annealing [12]. Veronica et al. studied the effect of laser shock strengthening on the residual stress of additive manufacturing stainless steel parts, and the results showed that laser shock strengthening reduced the tensile back stress by 247.61 MPa in the direction of X-Y construction [13]. Compared with the above methods, optimizing the residual stress by adjusting the grain characteristics of electroplated Cu can accurately control the residual stress from the microscopic level, which is a promising method. However, regulating the residual stress by controlling the microstructure of electroplated Cu has not been reported so far in the field of advanced electronic packaging.

It has been reported that nanotwinned Cu is a kind of interconnect material with excellent performance, and there is a strong relationship between the formation of nanotwin and the release of stress [14,15,16,17]. This means that it is possible to control the residual stress in electroplated Cu by controlling the structure of the nanotwin. In this paper, a method to regulate the residual stress of electroplated Cu by controlling the nanotwin directions was proposed.

## 2. Materials and Methods

### 2.1. Electroplating

The Cu layer was electroplated on a glass substrate with a 100 nm Ti adhesion layer and a 500 nm Cu seed layer. In order to ensure the integrity of the metal layer, the sputtering process was in the vacuum chamber of the magnetron sputtering instrument (VCT 300). An electrochemical workstation (VERSASTAT3) was used to generate a stable DC current, and the current density used for electroplating in this study was 12 mA/cm^2^ (ASD). The anode was a high-purity Cu sheet, and the cathode was a glass substrate with a metal seed layer. The 500 mL electroplating solution consisted of CuSO_4_·5H_2_O (100 g), NaCl (50 ppm), gelatin (50–300 ppm), and H_2_SO_4_ (10 g), with the rest being deionized water. The electroplating solution was stirred by magnetic stirring at a speed of 1000 rpm/min, and the electroplating process was carried out at room temperature.

### 2.2. Annealing

For the annealing process, the Cu samples were heated in a vacuum annealing furnace. The heating rate was 5 °C/min, the set temperature was 200 °C, and the holding time was 2 h. The vacuum pressure in the annealing furnace is maintained at 5 × 10^−4^ MPa during the heating process. Then, the samples were naturally cooled to room temperature in the furnace. To prevent oxidation, the samples were kept in a vacuum environment during heating and cooling.

### 2.3. Characterization

The microstructure of electroplated Cu was observed by a focusing ion beam (DB-FIB, Helios 5 CX). The acceleration voltage was 30 kV, and the observed current was 40 pA. The X-ray diffraction (XRD, Smart Lab SE) technique was used to determine the residual stress and preferred orientation of the sample. The speed of the X-ray scan was 5°/min, and the step size was 0.02°. In order to characterize the grain characteristics and the geometric necessity dislocation density of the sample, the electron backscattering diffraction (EBSD, Quanta 650) method was used. Before EBSD experiments, the surface of the sample was smoothed using FIB to remove the stress. EBSD was operated at a voltage of 20 kV and a step size of 0.12 µm. The EBSD data were calculated using Aztec Crystal software (version 2.1).

The residual stress was calculated using the classical sin^2^ψ-strain method based on XRD technology [18,19,20]. During the residual stress test, each sample was sequentially tilted at 0°, 15°, 27°, 31°, 35°, 39°, and 45° angles, and the diffraction peak of Cu_(311)_ was selected for measuring strain. The residual stress of Cu was calculated by Equation (1) [19]:

(1) $$\varepsilon_{\psi }=\frac{d_{\psi }-d_{0}}{d_{0}}=\left(\frac{1+\upsilon }{E}\right)\cdot \sin^{2}\left(\psi \right)\cdot \sigma -\left(\frac{2\upsilon }{E}\right)\cdot \sigma$$

where ε_ψ_ is the strain, and d_0_ was the (311) lattice spacing at ψ = 0, d_ψ_ is the (311) lattice spacing for every tilted ψ angle. υ and E were the Poisson’s ratio and Young’s modulus of Cu_(311)_, respectively, and σ is the residual stress of the Cu. The 2θ angle at each tilted angle can be obtained by using the parabolic fitting method, and then the Cu_(311)_ lattice spacing at each tilted angle was calculated based on Bragg’s law. Strain can be approximated as equal to (d_ψ_ − d_0_)/d_0_. Finally, the residual stress could be derived from the slope in the strain versus sin^2^ψ plot. In order to reduce the error, the residual stress of three identical samples is measured, and the average value is obtained.

## 3. Results and Discussion

### 3.1. Manipulating Nanotwin Directions Using Different Electroplating Conditions

**Figure 1:**
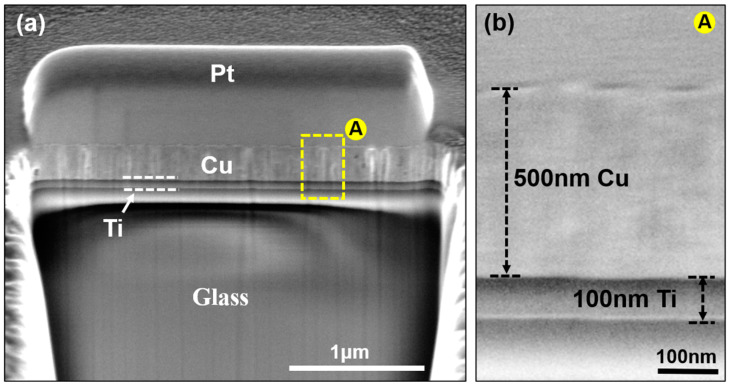
Microstructure of Ti-Cu seed layer sputtered on glass substrates: (**a**) FIB image; (**b**) SEM magnification image of area “A”.

In order to fabricate nanotwinned Cu, it is necessary to firstly sputter the seed layer with good quality. In this study, 100 nm Ti and 500 nm Cu were prepared on the surface of the glass substrate, as shown in Figure 1a. There is no gap in the contact interface between glass and Ti and between Ti and Cu, as shown in Figure 1b.

**Figure 2:**
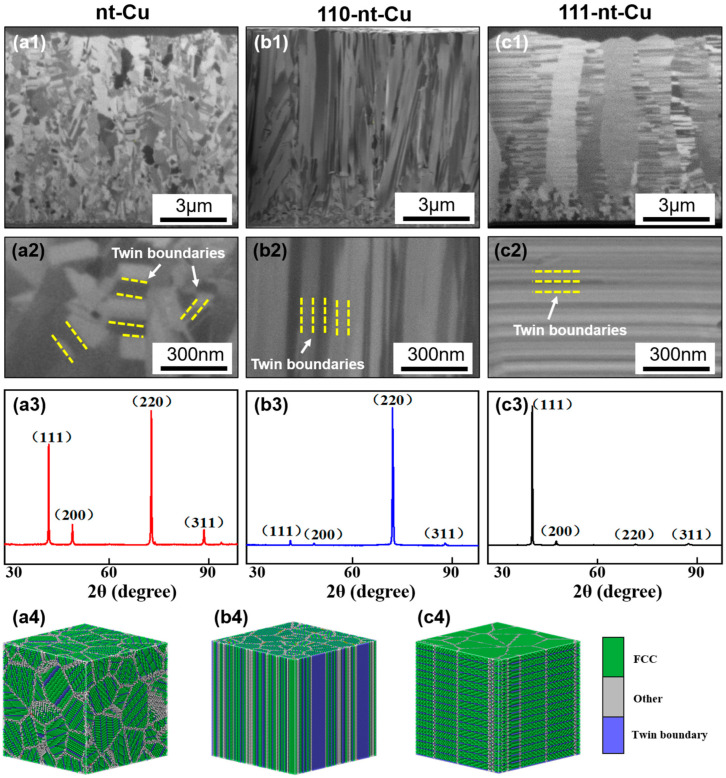
Cross-sectional FIB images of electroplated Cu and corresponding XRD results: (**a1**–**a3**) nt-Cu; (**b1**–**b3**) 110-nt-Cu; (**c1**–**c3**) 111-nt-Cu. (**a1**–**c1**) FIB images; (**a2**–**c2**) magnification image; (**a3**–**c3**) XRD results; (**a4**–**c4**) three-dimensional atomic arrangement model.

With different electroplating conditions, nanotwinned Cu with different orientations could be obtained. The FIB and XRD results of electroplated Cu under different electroplating conditions are shown in Figure 2. It can be found that three kinds of electroplated Cu have a large number of twin boundaries. According to XRD results, there is a significant difference in the orientation of the three kinds of electroplated Cu, among which the electroplated Cu with small grain size (Figure 2(a1)) presents a non-oriented state, and the crystal preferred orientations of the latter two are 110 (Figure 2(b1)) and 111 (Figure 2(c1)). For the convenience of distinction, in this paper, the three types of nanotwinned Cu are named as nt-Cu, 110-nt-Cu, and 111-nt-Cu, respectively. It is worth noting that the twin boundaries in nt-Cu are random, and the direction is not fixed, as shown in Figure 2(a2). However, the twin boundaries of 110-nt-Cu and the normal direction of the glass substrate are parallel, as shown in Figure 2(b2). 111-nt-Cu is composed of columnar grains with a large number of horizontal twins, as shown in Figure 2(c1,c2). This indicates that there is a high correlation between the nanotwin direction and the crystal preferred orientation. Based on the above analysis, the schematic diagram of the three-dimensional atom arrangement model of three different types of nanotwins is given, as shown in Figure 2(a4–c4). In summary, nanotwins with three different directions (non-directional, vertical, and horizontal) were successfully prepared under certain electroplating conditions.

Numerous studies have proved that additives are necessary for nanotwin formation [21,22,23]. Electroplated Cu without electroplating additives is usually composed of coarsely equiaxed grains with no preferred orientation. The basic electroplating solution in this study is an acidic CuSO_4_ solution, in which H_2_SO_4_ is used to increase the uniformity of the electroplating metal [24].

#### 3.1.1. 110-nt-Cu

Besides H_2_SO_4_, other additives could greatly influence the nanotwin feature. For the 110-nt-Cu electroplating solution, 50 ppm of NaCl was added into the based electroplating solution. The FIB results indicate that the introduction of Cl^−^ can induce the formation of the preferred orientation of (110). During the electroplating process, freely mobile Cl^−^ were preferentially adsorbed on the (110) crystal plane to promote the reduction of Cu^2+^ [25]. Since the growth direction of the electroplated Cu is parallel to the normal direction of the substrate, the rapidly growing (110) crystal plane thickens along the normal direction. At the beginning of electroplating, due to the inconsistent growth rates of different crystal planes, there would be significant residual stress between the grains. When the residual stress was too large, twinning behavior occurred, i.e., vertical twins with a Σ3 60° coherent grain boundary were formed (Figure 2(b2)).

#### 3.1.2. 111-nt-Cu

For 111-nt-Cu, gelatin can induce the formation of the preferred orientation of (111). A total of 50 ppm gelatin was added into the electroplating solution of 110-nt-Cu. The FIB results show that the introduction of gelatin can significantly change the preferred orientation and microstructure of electroplated Cu; that is, the original vertical nanotwin with (110) orientation was transformed into the horizontal nanotwin with (111) orientation (Figure 2(c2)). This may be related to the fact that gelatin is a large molecular weight inhibitor. It can be adsorbed onto the cathode surface to increase the probability of Cu atom misarrangement during deposition, thus promoting the formation of 111-oriented nanotwins. But gelatin is not the only condition for the formation of 111-nt-Cu. According to the experimental conclusions of Chen et al. [26], high-speed stirring of electroplating solution is also an important condition. In this study, a stirring speed of 1000 rpm/min was also implemented. Since the high-speed rotating electroplating solution produces a large shear stress on the surface where Cu is being deposited, the deposited metal layer has a large residual stress [23]. The formation of nanotwins is an important way to release residual stress [14]. In other words, increasing the shear stress is helpful for the formation of nanotwins. The macromolecular gelatin could also increase the shear stress between the high-speed flow electroplating solution and the Cu surface by increasing the viscosity of the electroplating solution. In addition, because Cu is a face-centered cubic metal, the twin plane is the (111) crystal plane. Therefore, under the combined influence of high-speed stirring of electroplating solution and gelatin, horizontal nanotwins with high percentage (111) orientation were formed.

#### 3.1.3. nt-Cu

As the gelatin content increased to 200 ppm, the microstructure and orientation of electroplated Cu changed. The original horizontal twins with (111) orientation were transformed into grains with no obvious preferred orientation and irregular twin direction, that is, ordinary nt-Cu, as shown in Figure 2(a2). This suggests that too much gelatin would change the current 111-oriented nanotwin status.

In summary, by controlling electroplating conditions (concentration of Cl^−^ and gelatin, stirring speed), nanotwins with three different directions (non-directional, vertical, and horizontal) can be fabricated.

**Figure 3:**
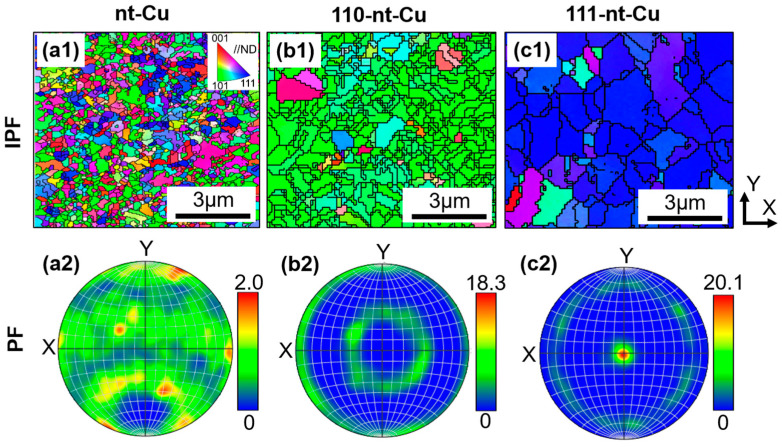
Plan-view EBSD images of electroplated Cu results: (**a1**,**a2**) nt-Cu; (**b1**,**b2**) 110-nt-Cu; (**c1**,**c2**) 111-nt-Cu. (**a1**–**c1**) Inverse pole figures; (**a2**–**c2**) Pole figures of {111}.

In order to further validate the preferred orientation of the three types of nanotwins above, EBSD experiments on the top plane of electroplated Cu were carried out. Figure 3 shows the inverse pole figures (IPFs) and pole figures (PFs) of electroplated Cu with three different nanotwin directions. In the IPF, the green, blue, and red grains represent the preferred orientations of (110), (111), and (100), respectively. According to the IPFs (Figure 3(a1–c1)), the grains of 110-nt-Cu and 111-nt-Cu belong to the green and blue categories, respectively. This indicates that the preferred orientation of 110-nt-Cu and 111-nt-Cu is (110) and (111), respectively. The IPFs of nt-Cu are composed of grains of various colors, indicating that there is no obvious preferred orientation in the sample. The same results could be obtained in the corresponding PFs (Figure 3(a2–c2)). Therefore, the results obtained by EBSD and XRD (Figure 2(a3–c3)) are consistent.

### 3.2. Residual Stress of Nanotwins During Electroplating

**Figure 4:**
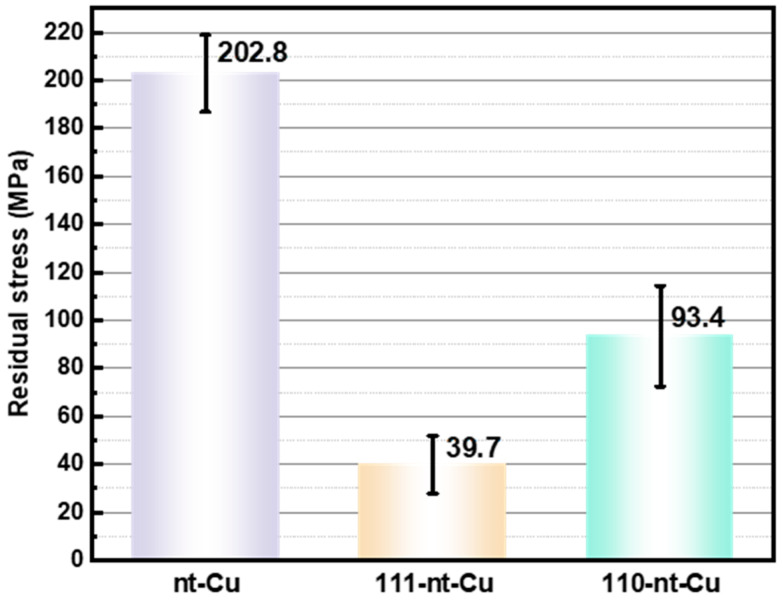
Residual stress of electroplated Cu with different nanotwin directions.

Figure 4 shows the residual stresses of electroplated Cu with three different nanotwin directions, which are calculated by sin^2^ψ-strain method-based XRD technology. Specifically, the residual stresses of nt-Cu, 111-nt-Cu, and 110-nt-Cu are 202.8 MPa, 39.7 MPa, and 93.4 MPa, respectively. 111-nt-Cu has the lowest residual stress after electroplating. The value of the residual stress is highly related to the direction of the nanotwins.

For 111-nt-Cu, the formation of high-density horizontal nanotwins can release a large amount of microscopic stress, so the residual stress inside the final electroplated Cu is low. The nanotwin direction of 110-nt-Cu is vertical so that the residual stress in the horizontal plane cannot be released and will be retained until the end of electroplating. It is worth noting that the twin density of 110-nt-Cu is much lower than that of 111-nt-Cu, as shown in Figure 2(b2,c2).

For nt-Cu, the higher residual stress is related to two main factors: grain size and twin direction. The average grain size of nt-Cu is only a few hundred nanometers (Figure 3(a2)), so there are a large number of common high-angle boundaries inside the sample. Because the common high-angle boundary belongs to the region of atomic dislocation, the lattice distortion is serious, causing a lot of microscopic stress. In addition, although there are a large number of nanotwins in the grain of nt-Cu, the residual stress inside the grain cannot be released in the horizontal direction since the direction of the twins is random. Therefore, grain size and twin direction both lead to the high residual stress of nt-Cu.

### 3.3. Changes of Microstructure and Residual Stress of Nanotwin During Annealing

**Figure 5:**
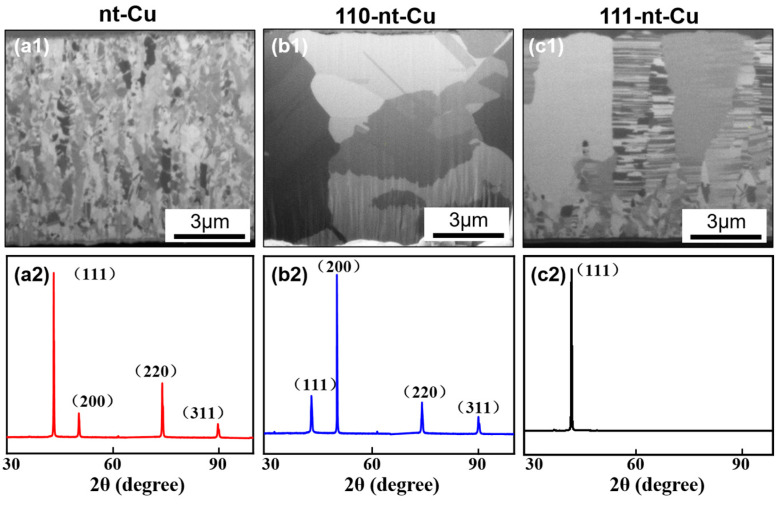
FIB and XRD results of electroplated Cu results after annealing at 200 °C for 2 h: (**a1**,**a2**) nt-Cu; (**b1**,**b2**) 110-nt-Cu; (**c1**,**c2**) 111-nt-Cu. (**a1**–**c1**) FIB results; (**a2**–**c2**) XRD results.

After electroplating, the three types of nanotwins were annealed at 200 °C for 2 h. Figure 5 shows the microstructure and XRD results.

**Figure 6:**
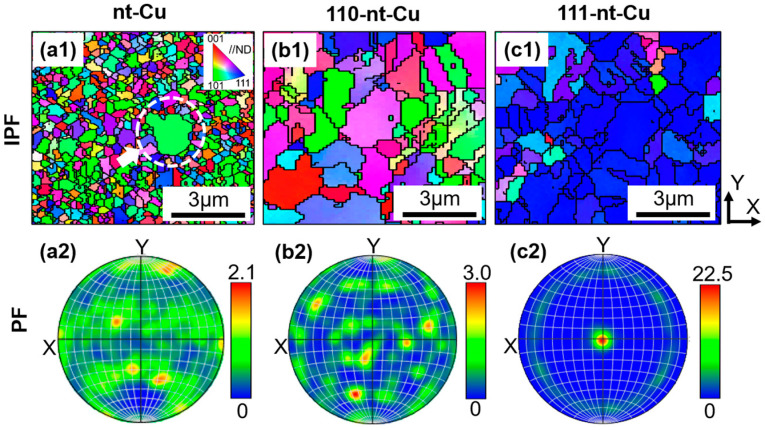
Plan-view EBSD images of electroplated Cu results after annealing at 200 °C for 2 h: (**a1**,**a2**) nt-Cu; (**b1**,**b2**) 110-nt-Cu; (**c1**,**c2**) 111-nt-Cu. (**a1**–**c1**) Inverse pole figures; (**a2**–**c2**) Pole figures of {111}.

It can be observed that 110-nt-Cu had undergone significant recrystallization behavior during the annealing process. The original vertical nanotwins became a large recrystallized grain, as shown in Figure 5(b1). The corresponding XRD results (Figure 5(b2)) show that the original (220) diffraction peak drops sharply while the (200) diffraction peak increases significantly, meaning that the 100-oriented grains grew rapidly during the annealing process. This is attributed to the thermal stress created during annealing. Under thermal stress conditions, since the (100) plane has the lowest strain energy [27], 100-oriented grains grew to reduce the strain energy of the electroplated Cu. The PFs and IPFs also prove that the 100-oriented grains of 110-nt-Cu increased during annealing, as shown in Figure 6(b1,b2). This is similar to the results of Wu et al. [28]. In their study, during the annealing treatment at 200 °C, large and medium-sized recrystallized grains started to grow from the transition layer closest to the substrate and gradually engulfed 110-oriented vertical nanotwins.

For 111-nt-Cu, the high-density horizontal nanotwins remained after annealing treatment, and no obvious recrystallization occurred. This is due to the presence of a large number of low-energy twins. Its preferred orientation after annealing is still a strong (111) preferred orientation, which is verified in the corresponding XRD and PFs, as shown in Figure 5(c2) and Figure 6(c2). It is worth noting that the columns composed of high-density horizontal nanotwins appeared to be wider after annealing than before annealing (Figure 2(c1)), indicating that some horizontal nanotwins merged with each other. It inevitably reduced lattice distortion and dislocation density.

The grain size and grain characteristics of nt-Cu after annealing treatment do not seem to change, as shown in Figure 5(a1). In addition, XRD and EBSD results (Figure 5(a2) and Figure 6(a1)) show that the annealed grains still have no obvious preferred orientation. This may be caused by the presence of a large number of nanotwins in nt-Cu. The twin boundary energy is only one-tenth of the ordinary high-angle boundary energy [29]. Thus, the grains are not easy to recrystallize violently during annealing. Therefore, nt-Cu has high thermal stability. In addition, the IPF (Figure 6(a1)) of nt-Cu after annealing shows a large grain size, which indicates that nt-Cu has a weak local recrystallization behavior, but the energy provided by the annealing process in this study is not enough to produce significant recrystallization. It is possible that further recrystallization of nt-Cu can occur by extending the annealing time or increasing the annealing temperature, which requires long-term research.

**Figure 7:**
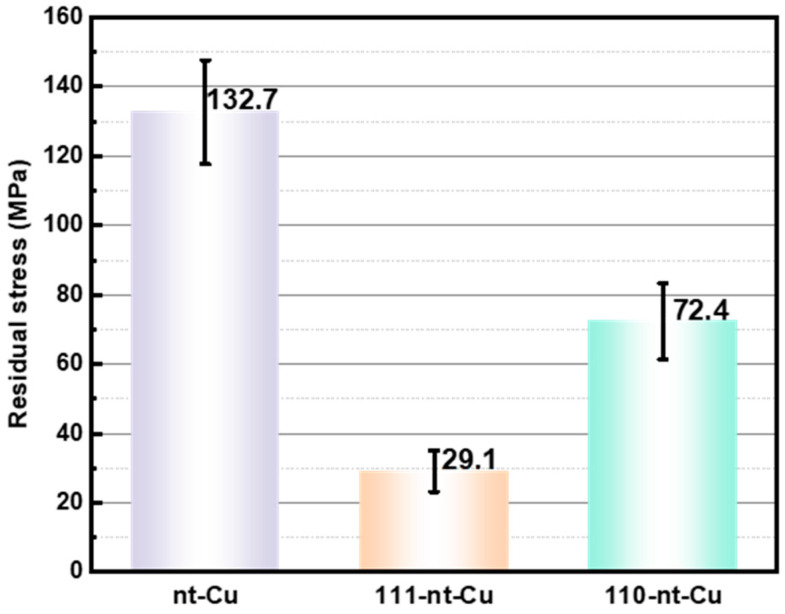
Residual stress of electroplated Cu with different nanotwin directions after annealing at 200 °C for 2 h.

Figure 7 shows the residual stress of electroplated Cu with three different nanotwin directions after annealing. Specifically, the residual stresses of annealed nt-Cu, 111-nt-Cu, and 110-nt-Cu are 132.7 MPa, 29.1 MPa, and 72.4 MPa, respectively. Compared with before annealing, the residual stress of nt-Cu, 111-nt-Cu, and 110-nt-Cu after annealing treatment decreased by 34.6%, 26.7%, and 22.5%, respectively, as shown in Table 1. The residual stress of 111-nt-Cu decreased the lowest percentage after annealing, which may be related to its relatively stable microstructure. However, the residual stress of 111-nt-Cu after annealing is only 29.1 MPa, which is a very low stress in electroplated Cu [20]. This proves that the residual stress of electroplated Cu on the surface of a glass substrate can be significantly reduced by a reasonable electroplating process combined with an annealing treatment. However, it is necessary to pay attention to the choice of annealing temperature, because too high annealing temperature will cause plastic deformation of electroplated Cu, resulting in an increase in residual stress, which is not expected.

Based on the discussion above, 110-nt-Cu had a violent recrystallization, whereas 111-nt-Cu and nt-Cu had only a slight recrystallization. The annealing process reduced the residual stress of electroplated Cu, and the residual stress of 111-nt-Cu was still the lowest after annealing.

### 3.4. Mechanism of the Residual Stress Change Based on Geometric Necessity Dislocation Method

**Figure 8:**
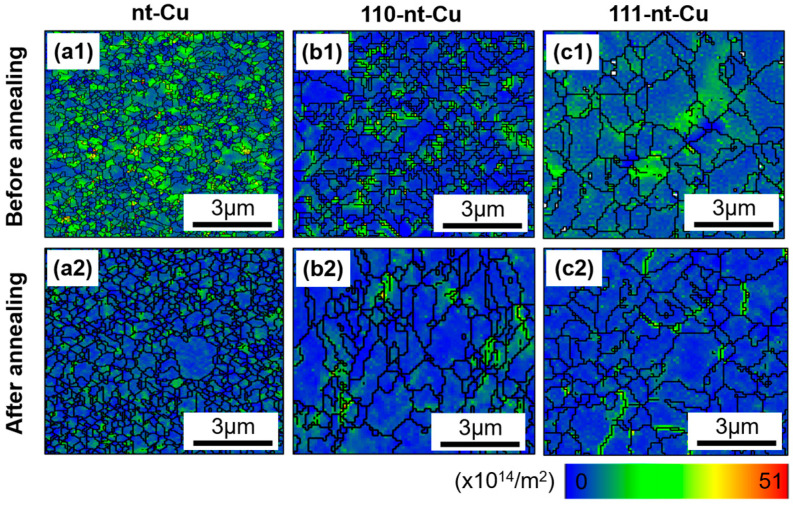
Distribution map of geometric necessity dislocation of electroplated Cu: (**a1**,**a2**) nt-Cu; (**b1**,**b2**) 110-nt-Cu; (**c1**,**c2**) 111-nt-Cu. (**a1**–**c1**) Before annealing; (**a2**–**c2**) after annealing.

In the process of annealing, the three kinds of nanotwinned Cu had different degrees of crystallization behavior, and the corresponding grain characteristics also changed. The change in grain characteristics is the direct reason for the change in residual stress. However, it is difficult to find a direct correlation between the microstructure characteristics and the change of residual stress. Given that, here we develop a novel characterization of residual stress on the microstructure, namely geometric necessity dislocation. Geometric necessity dislocation represents the statistical result of the small misorientation between and within the grains, and the average dislocation density of the sample can be obtained by calculating the EBSD data. Geometric necessity dislocation in a sense represents the degree of lattice distortion, which in turn represents the distribution and magnitude of microscopic stress. Figure 8 shows the geometric necessity dislocation distribution maps of three kinds of electroplated Cu before and after annealing. The blue-to-red rainbow indicates the increasing geometric necessity dislocation density. In other words, the longer the wavelength of the color, the greater the geometric necessity dislocation density. It is obvious that the blue region of the three electroplated Cu increased after annealing treatment, demonstrating that the geometric necessity dislocation density of the sample decreased.

**Figure 9:**
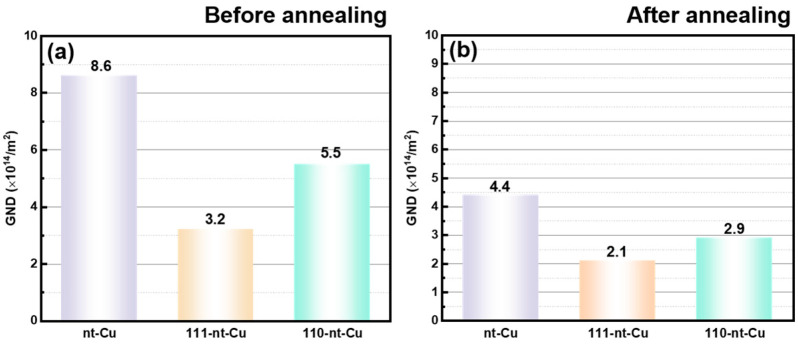
Geometric necessity dislocation density of electroplated Cu: (**a**) Before annealing; (**b**) after annealing.

Figure 9 shows the geometric necessity dislocation density statistic results of nanotwinned Cu before and after annealing. Before annealing, the average geometric necessity dislocation densities of nt-Cu, 111-nt-Cu, and 110-nt-Cu are 8.6 × 10^14^/m^2^, 3.2 × 10^14^/m^2^, and 5.5 × 10^14^/m^2^, respectively, while after annealing, their average geometric necessity dislocation densities are 4.4 × 10^14^/m^2^, 2.1 × 10^14^/m^2^, and 2.9 × 10^14^/m^2^, respectively. It is obvious that there is also a close relationship between the geometric necessity dislocation density value of electroplated Cu and the nanotwin direction, and this trend seems to be consistent with the relationship between the nanotwin direction and the residual stress. That is, compared with 110-nt-Cu and nt-Cu, 111-nt-Cu has the lowest geometric necessity dislocation density after electroplating and annealing.

As shown in Table 2, the average geometric necessity dislocation density of nt-Cu, 111-nt-Cu, and 110-nt-Cu decreased by 48.8%, 34.3%, and 47.3%, respectively, compared with that before annealing, indicating that the lattice distortion and small orientation difference introduced in the electroplating process decreased. The decrease in geometric necessity dislocation density is caused by recrystallization behavior, because the recrystallization behavior of grains during annealing can cause grain boundary migration and sub-grain merger, resulting in the annihilation of dislocation and the reduction of grain boundaries. The reduction of dislocation density would inevitably reduce the degree of lattice distortion and then reduce the residual stress in electroplated Cu. Therefore, it can be concluded that the reduction in residual stress of electroplated Cu after annealing treatment can be attributed to the decrease in geometric necessity dislocation density.

## 4. Conclusions

In this paper, a method to control the residual stress of electroplated Cu on the glass substrate surface was proposed for the first time by manipulating nanotwin directions. The mechanism of the change of microstructure and residual stress during electroplating and annealing processes was investigated systematically. The specific research conclusions are as follows:

(1) By controlling electroplating conditions (concentration of Cl^−^ and gelatin, stirring speed), nanotwins with three different directions (nt-Cu, 110-nt-Cu, 111-nt-Cu) can be fabricated.
(2) The residual stresses of nt-Cu, 111-nt-Cu, and 110-nt-Cu after electroplating are 202.8 MPa, 39.7 MPa, and 93.4 MPa, respectively. 111-nt-Cu has the lowest residual stress after electroplating. The value of the residual stress is highly related to the direction of the nanotwins.
(3) During annealing at 200 °C, 110-nt-Cu had a violent recrystallization, whereas 111-nt-Cu and nt-Cu had only a slight recrystallization. The annealing process reduced the residual stress of electroplated Cu, and the residual stress of 111-nt-Cu was still the lowest after annealing.
(4) There is a strong relationship between geometric necessity dislocation density and residual stresses. The reduction in residual stress of electroplated Cu during annealing treatment can be attributed to the decrease in geometric necessity dislocation density.

## Figures and Tables

**Table 1:** Residual stress of electroplated Cu with different nanotwin directions before and after annealing.

	**nt-Cu**	**111-nt-Cu**
Before annealing (MPa)	202.8	39.7
After annealing (MPa)	132.7	29.1
Percentage reduction	34.6%	26.7%

**Table 2:** Geometric necessity dislocation density of electroplated Cu with different nanotwin directions before and after annealing.

	nt-Cu	111-nt-Cu
Before annealing (×10^14^/m^2^)	8.6	3.2
After annealing (×10^14^/m^2^)	4.4	2.1
Percentage reduction	48.8%	34.3%

## Data Availability

The data are included in the article. If further information is required, the data are available on request from the authors.
